# Functional expression and localisation of HOPS/TMUB1 in mouse lens

**DOI:** 10.1042/BSR20203998

**Published:** 2021-02-12

**Authors:** Daniela Bartoli, Danilo Piobbico, Marilena Castelli, Stefania Pieroni, Damiano Scopetti, Simona Ferracchiato, Nicola Di-Iacovo, Carlo Cagini, Giuseppe Servillo, Maria Agnese Della-Fazia

**Affiliations:** Department of Medicine and Surgery, Piazzale L. Severi 1, University of Perugia, Perugia, Italy

**Keywords:** differentiation, HOPS/TMUB1, lens, proliferation, UBL modifier

## Abstract

Transparency represents the functional phenotype of eye lens. A number of defined steps including quiescence, proliferation, migration and cell differentiation culminates in cell elongation and organelle degradation, allowing the light to reach the retina. HOPS (Hepatocyte Odd Protein Shuttling)/TMUB1 (Trans Membrane Ubiquitin-like containing protein 1) is a nucleo-cytoplasmic shuttling protein, highly expressed both *in vivo* and *in vitro* proliferating systems, bearing a *ubiquitin-like domain*. The present study shows HOPS expression during the phases of lens cell proliferation and fiber differentiation, and its localisation in lens compartments. In lens, HOPS localises mainly in the nucleus of central epithelial cells. During mitosis, HOPS/TMUB1 shuttles to the cytoplasm and returns to the nucleus at the end of mitosis. The differentiating cells share distinct HOPS/TMUB1 localisation in transitional zone depending on the differentiation phases. HOPS/TMUB1 is observed in lens cortex and nucleus. Here, it is attached to fibers, having a structural function with crystallin proteins, probably acting in the ubiquitin–proteasome system.

## Introduction

HOPS (Hepatocyte Odd Protein Shuttling) or TMUB1 (Trans Membrane Ubiquitin-like containing protein 1), —hereafter HOPS—is a shuttling protein with a *ubiquitin-like* domain (UBL), three *transmembrane* domains and a *proline-rich* domain. In the nucleus of quiescent cells, HOPS appears mainly as speckles and dots. *Hops* cDNA translates three different proteins with distinct molecular weights. The analysis of *Hops* coding sequence showed a first methionine encoding for a 245-amino acid (Aa) isoform of 27 kDa (long-HOPS, l-HOPS) and an alternative methionine, at 55 Aa, acting as a second starting point, translating a 21-kDa isoform (short-HOPS, s-HOPS). A signal-peptide site at the N-terminus—a putative cleavage site—defines the 24-kDa intermediate form (intermediate-HOPS, i-HOPS) [1].

Originally, HOPS gene was identified as expressed in liver regeneration induced by partial hepatectomy (PH). During the first steps of proliferation HOPS migrates from nucleus to cytoplasm to be back into the nucleus at the end of mitosis [2]. Many growth factors and molecules controlling proliferation of residual hepatocytes affect HOPS export. Epidermal growth factor (EGF) and cyclic AMP (cAMP) play an important role in HOPS nucleus/cytoplasm shuttling. HOPS export to the cytoplasm is chromosome region maintenance-1 (CRM1)-mediated [2]. In tumour cells, HOPS overexpression acts as a suppressor of proliferation [2]. HOPS localises in centrosome and its depletion leads to supernumerary centrosomes, abnormal spindles, multinucleated cells, and genomic instability in NIH-3T3 cells. HOPS depletion drastically reduces mitotic figures and arrests the cells in G_0_/G_1_ phase [3]. Moreover, it has been demonstrated that HOPS affects p19^Arf^ stability acting as a bridging protein in nucleophosmin (NPM)–p19^Arf^ interaction [4]. Recently, HOPS has been identified as a regulator of cytoplasmic p53 function and fate. HOPS is involved in p53 stabilisation, p53-mediated mitochondrial apoptosis and p53 nuclear import. This evidence suggests that HOPS acts as potential tumour suppressor by its ubiquitin-like domain [5–7].

**Figure 1:**
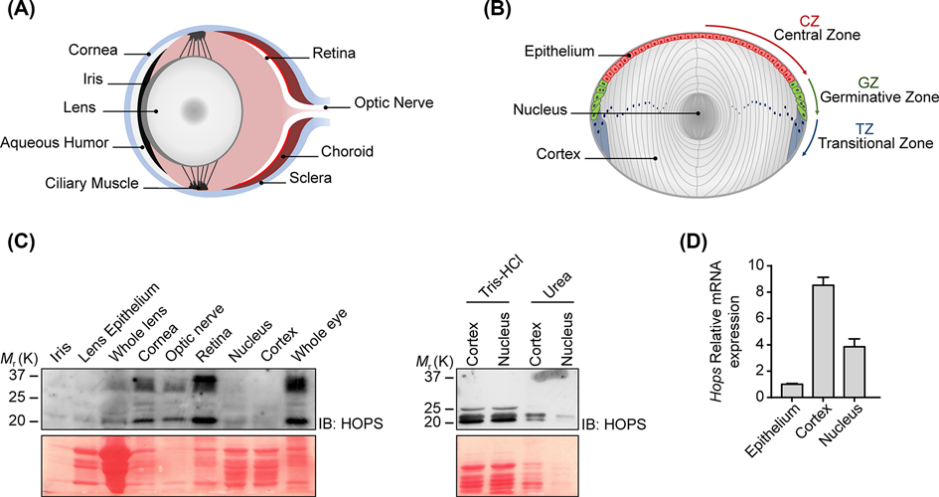
Mouse eye and lens structure (**A,B**) Mouse eye (A) and lens (B) schematic overview with main components indication. (**C**) WB analysis of HOPS protein expression in eye and lens compartments (left). The fibers proteins of lens cortex and nucleus obtained upon Tris/HCl (lanes 1, 2) and urea treatment (lanes 3, 4) evaluated by WB (right). Ponceau S staining was used to evaluate protein amount. Images are representative of one mouse eye proteins. (**D**) qPCR showing *Hops-mRNA* in lens epithelium, cortex and nucleus. mRNA in lens epithelium was assumed as 1 and samples were normalised to *Gusb* gene. Representative images are shown.

The lens is a transparent, avascular and innervation devoid organ, enclosed in a collagenous capsule, sited between cornea and retina (Figure 1A). In the eye, it guarantees transparency and refractive power. The lens is exposed to environmental insults such as U.V. light, smoke and other agents that can damage its structure, leading to cataract formation [8,9].

The lens is considered a very good and simple experimental model to study the protein connections, simultaneously in cellular quiescence, proliferation, differentiation and apoptosis systems [10–12].

The lens epithelium is subdivided into three distinguished zones (Figure 1B): the anterior central zone (CZ), consisting of quiescent and undifferentiated cells with few organelles; the germinative zone (GZ)—a narrow zone underlying the ciliary body—defined by proliferating cells; the transitional zone (TZ)—near the equator plane—in which, lens postmitotic epithelial cells begin to differentiate in lens fibers, reserving transcription and translational activity [13].

Here, using the lens as physiological model, we observed that HOPS localisation changes in different lens compartments relating to cell cycle and differentiation, and we evaluated its role in terminal phase of fibers differentiation. We assume that HOPS might play an important role in controlling proliferation and differentiation of lens epithelial cell (LEC).

## Methods

### Animals

One-month-old SVJ 129 mice (Charles River Laboratories, Milan, Italy) were housed in animal house facility of University of Perugia. The mice were maintained in a pathogen-free barrier area on standard 12-h/12-h light/dark cycle with *ad libitum* food and water in ∼25°C and 40–60% humidity. Untreated and treated animals were killed by cervical dislocation. For EGF treatment, mice were killed at 0, 30 and 60 min after intraperitoneal injection of 10 μg/g body weight of EGF (Sigma–Aldrich, St. Louis, MO, U.S.A.). For each experimental point, the lenses were collected and O.C.T.-embedded for immunofluorescence analysis [14,15].

### Isolation of lens epithelium

Eyes were explanted and quickly included in Tissue-Tek® O.C.T. Compound (Sakura Finetek, Torrance, CA, U.S.A.). The eye was sectioned by coronal incision in the sclera. Retina and vitreous were discarded, the posterior side of the lens was exposed and the suspensory ligaments were severed to release the lens. For lens epithelium whole mounts, the lens was placed with capsular side down on a Superfrost-Plus™ slide (Thermo Fisher Scientific, Waltham, MA, U.S.A.). Fiber mass with posterior capsule was discarded. The slides were dried at 30°C and stored at −80°C [10,16].

### Primary LECs

For primary LEC cultures, the epithelia explants with the capsular side downwards, were placed in 35-mm Petri dishes containing DMEM medium with Foetal Bovine Serum (FBS, EuroClone, Milan, I). LECs were placed in a 37°C incubator, 5% CO_2_ for 14 days, to allow epithelial cells to spread to Petri dishes. The preparations were cultured until epithelial cells recolonised the cell-denuded areas of the lens capsule and migrated from the lens capsule on to the dish. At confluence, cells were collected and 1 × 10^6^ cells were plated in 60-mm dish in differentiating medium, containing 100 ng/ml of basic fibroblast growth factor (b-FGF; Sigma–Aldrich). The culture medium was changed every 3 days until the monolayer confluence [17,18].

### Histological analyses and immunofluorescence

Lens epithelium whole mount and lens cryosection (7 μm) were performed as previously described [2]. Anti-vimentin and anti-γ-tubulin (Sigma–Aldrich) primary antibodies and Cy3 goat anti-rabbit (H+L) and Alexa 488 goat anti-mouse (H+L) secondary antibodies (Thermo Fisher Scientific) were used. DNA was DAPI stained (Sigma–Aldrich). Images were captured with a Zeiss Axioplan fluorescence microscope controlled by a Spot 2-cooled camera (Diagnostic Instruments, MI, U.S.A.).

### Cell proliferation analysis

Bromodeoxyuridine (BrdU; Sigma–Aldrich) was intraperitoneally injected in mice at a concentration of 1.5 mg/30 g body weight. After 1 h, mice were killed and lens epithelium whole mount was isolated and treated as previously described [2]. Proliferating cells were revealed with anti-BrdU antibody (Abcam, Cambridge, U.K.) and Alexa 488 goat anti-mouse (H+L) (Thermo Fisher Scientific) as secondary fluorescent antibody [19].

### Western blot analyses

Whole eye and dissected iris, cornea, retina, optic nerve, lens, lens epithelium, cortex and nucleus were immediately homogenised in Laemmli 1× buffer and boiled at 95°C for 5 min for protein analysis. The samples were sonicated and the supernatants stored at −80°C. Cortex and nucleus were separated from lens mass and homogenised in Tris/HCl 10 mM pH 7.4 + PMSF + PIC (Sigma–Aldrich) and sonicated. Then the samples were centrifuged at 100000×***g*** for 10 min at 4°C. The resulting pellets were resuspended in the same buffer and centrifuged at 10000×***g*** for 10 min at 4°C. The pellets were then dissolved in Urea 7 M (Sigma–Aldrich) in 10 mM Tris/HCl pH 7.4 and centrifuged twice at 100000×***g*** for 10 min at 4°C. The final pellets were dissolved in Tris/HCl pH 7.4 10 mM, sonicated and stored at −80°C [20].

Extracted proteins were subjected to Western blot analysis with anti-HOPS polyclonal antibody [2] and the appropriate HRP-conjugated secondary antibody (Bio-Rad, Hercules, CA, U.S.A.) and visualised with ECL (GE Healthcare Life Sciences, Little Chalfont, U.K.).

### RNA extraction and qPCR

RNA extraction and qPCR were performed as previously described [21,22]. Total RNA was extracted from lens epithelium, cortex and nucleus using TRIzol® reagent (Thermo Fisher Scientific) according to the manufacturer's instructions. cDNA was reverse-transcribed from 1 μg of RNA using the iScript™ kit (Bio-Rad). qPCR was performed by SYBR®Green qPCR Master Mix (Thermo Fisher Scientific). Primer sequences used for *Hops* detection are: *Hops* Forward 5′-GCCTCAGGACACCATTGG-3′; *Hops* Reverse 5′-CTAGCAGTTGACCTTGGTAGATG-3′. The relative amount of mRNA was normalised to *Gusb* gene.

### Statistical analyses

Analyses were performed using the Excel software and presented as means ± SEM. Images are representative of three experiments at least performed in triplicate.

## Results

### HOPS is expressed in lens epithelium

HOPS is expressed in all tissues examined [1]. So far, HOPS in the eye has been detected only in retina (Protein Atlas, https://www.proteinatlas.org). To evaluate and verify its presence in all lens components, we dissected and extracted proteins from the eye compartments and analysed HOPS protein expression through Western blotting analysis. HOPS expression was investigated in: iris, lens epithelium and whole lens, cornea, optical nerve, retina, lens nucleus, cortex and whole eye. Except for lens nucleus and cortex, we found the three HOPS isoforms in all the compartments examined (Figure 1C left). To assess whether HOPS absence was determined by technical aspects—due to difficult extraction of fiber proteins, attributable to the intricate intertwine between the proteins in the fibrous structure—or to lack of HOPS in the fibers, we performed protein extraction from lens nucleus and cortex using high salt concentration and strong chaotropic agent. The data obtained indicated that HOPS is present in lens fibers and is tightly linked to its structure [20] (Figure 1C right).

In addition, qPCR analysis confirmed the presence of *Hops* transcripts in the epithelium, cortex and nucleus lens (Figure 1D).

### HOPS localisation in different compartments of the lens cells

Once HOPS expression in the eye compartments was validated, we focussed our attention on studying HOPS localisation in different zones of the lens cells. As described above the lens represents a very interesting experimental model to analyse, in a single structure, cell quiescence, proliferation and differentiation. Because HOPS shuttles from nucleus to cytoplasm and *vice versa*, depending on cell cycle progression and stress stimuli, we analysed HOPS localisation in different regions of lens in relation to proliferating and/or differentiating areas.

**Figure 2:**
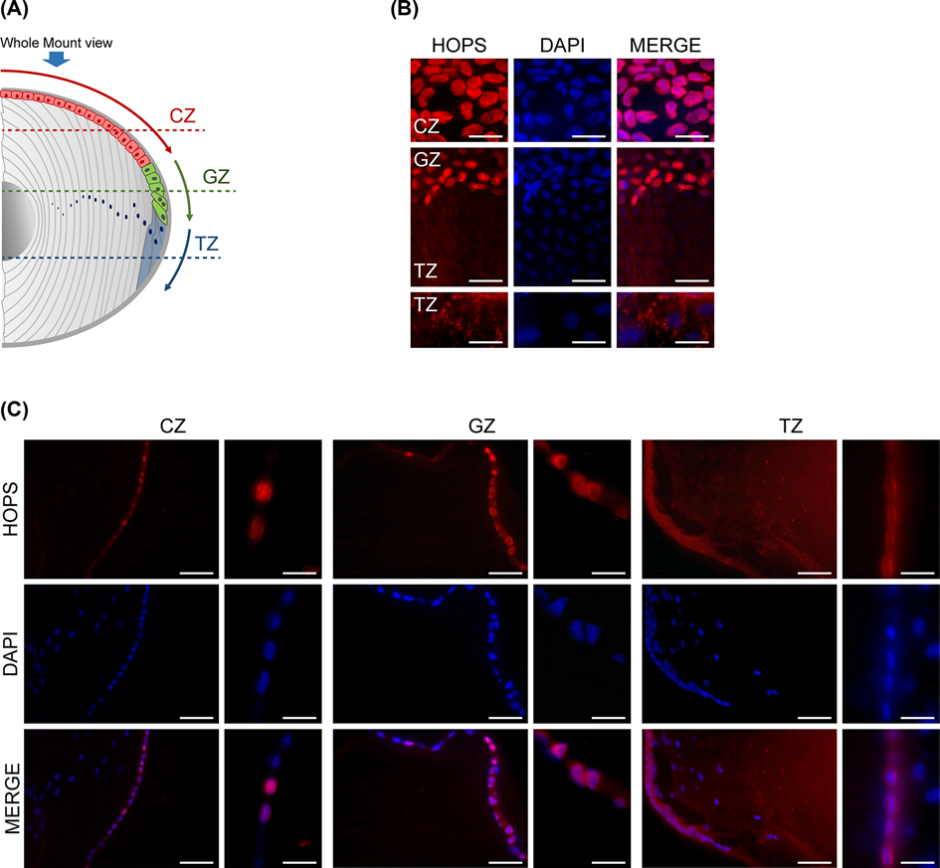
HOPS localisation in mouse lens epithelium (**A**) Graphical overview of mouse lens transverse sections (dashed lines) and whole-mount view (blue bold arrow) displayed in (**B,C**). (B) Lens epithelium whole-mount showing HOPS (red) cellular localisation. Nuclei were DAPI stained (blue). Bars, 10 μm. (C) Lens transverse sections at the CZ, GZ and TZ evidenced HOPS localisation (red) in the lens epithelium. Nuclei were DAPI stained (blue). For each zone, images at two magnifications are shown. Bars, 10 μm (left) and 5 μm (right). Merged images are shown. Representative images are shown.

HOPS distribution was analysed in whole mount epithelium. HOPS appears mainly in nucleus in quiescent cells of the central area. In GZ (Figure 2A), characterised by the presence of proliferating cells, HOPS assumes a perinuclear and cytoplasmic localisation, while, in TZ, HOPS is mostly cytoplasmic (Figure 2B).

The three different regions of the lens epithelium were analysed by transverse cryosections observation. In CZ, cells are quiescent and HOPS is highly expressed in the nucleus. Moving towards GZ we observed that HOPS is mainly localised in the perinuclear zone. Proceeding to TZ, where the differentiation of cells into fibers differentiation takes place, the localisation of HOPS is mainly cytoplasmic (Figure 2C).

### Identification of GZ by BrdU

To assess HOPS localisation in the lens epithelium and to better identify the mitotically active cell population associated to GZ, we injected BrdU in the mice as marker for replicating cells, before proceeding to dissection. Indeed, the mitotic index in adult lens is very low and GZ is restricted in a small district near the equatorial zone [13].

**Figure 3:**
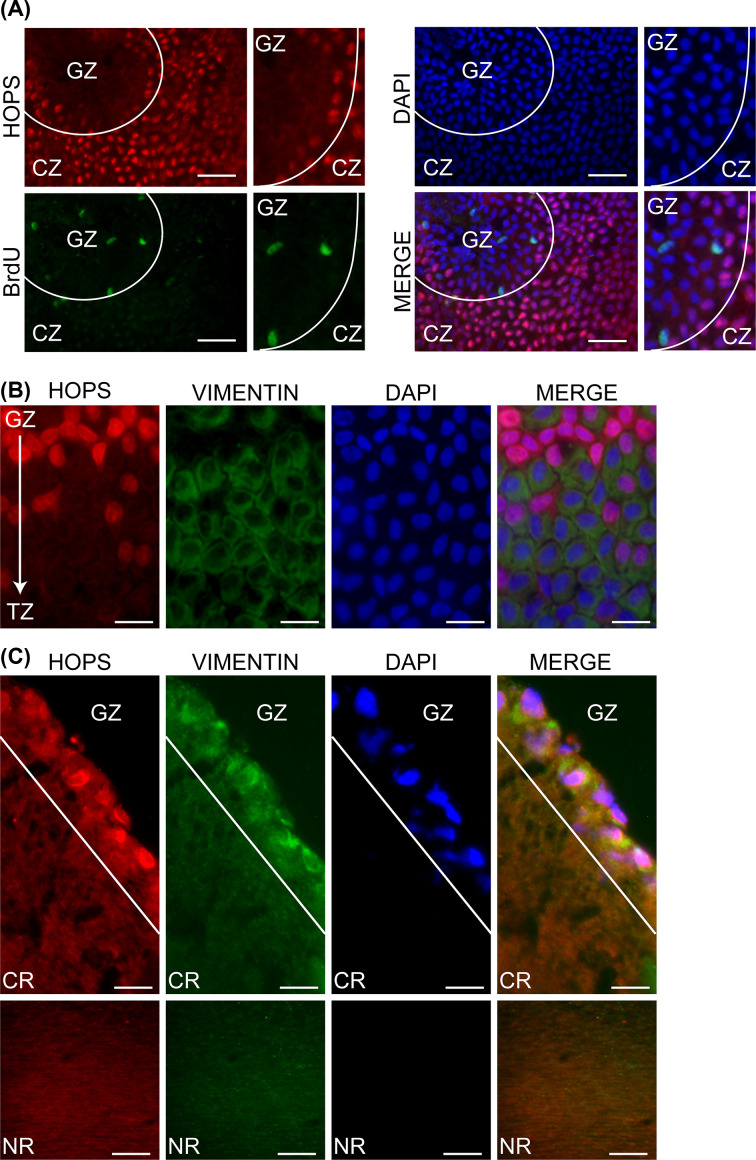
HOPS localisation in nuclear and cortical regions of the lens epithelium (**A**) HOPS (red) cellular localisation in whole-mount lens epithelium specimens of BrdU treated mice. BrdU (green) was used as proliferation marker to discriminate GZ from CZ. Nuclei were DAPI stained (blue). Bars, 10 μm. For each panel an image insight is reported on the right. (**B**) HOPS cellular localisation (red) observed in whole-mount lens epithelium. Vimentin (green) was used as marker to identify TZ. Nuclei were DAPI stained (blue). Bars, 5 μm. (**C**) Lens transversal sections at GZ were analysed for HOPS localisation (red) in nuclear (NR) and cortical (CR) regions. Vimentin (green) was used to identify cells layer and fibers of the lens epithelium. Nuclei were DAPI stained (blue). Bars, 5 μm. Merged images are reported. Representative images are shown.

In GZ of whole mount specimens, HOPS is mainly localised in the cytoplasm, according to the proliferative state of the cells. The analysis of GZ with BrdU positive cells identifications showed a diffuse localisation of HOPS, mainly perinuclear and cytoplasmic, concerning to the specific cell cycle phase (Figure 3A).

The data obtained confirmed that, at the beginning of the proliferative program, HOPS progressively leaves the nucleus to migrate to the cytoplasm while in proliferating cells HOPS resides in the cytoplasm.

### Identification of TZ by vimentin

Vimentin is an abundant intermediate filament protein of lens epithelium and superficial cortex whose distribution and structure have been shown to be zone-specific [23].

Vimentin was used to identify germinative and transition zone in the lens epithelium. In GZ, Vimentin takes the form of defined basket-like structures that persist in the TZ median cells. These structures are no longer visible in the sidelong cells of TZ [14].

The obtained data, analysing epithelium whole mount, show that in the GZ, HOPS assumes a perinuclear localisation, while in the TZ it becomes predominantly cytoplasmic (Figure 3B).

Moving from GZ towards the equatorial edge, epithelial dividing cells begin the differentiating program into fiber cells. HOPS detection at equatorial rim showed a group of proliferating cells, where HOPS is retained in cytoplasm. Furthermore, we used vimentin as marker of lens cell differentiation. In the same fields, Vimentin showed the classical fiber-like aspect, indicating a cell switch towards differentiation [24].

In GZ, the analysis carried out in transverse sections confirms perinuclear and cytoplasmic HOPS localisation and highlights its colocalisation with vimentin at fibers in cortex and nucleus regions (CR and NR). (Figure 3C). The data obtained from immunofluorescence analysis, qPCR and Western blotting, tightly confirm the presence of HOPS also at the level of the cortex fibers and the nucleus of the lens (Figure 1C,D).

### EGF treatment

Since HOPS localisation in lens is determined by cell cycle phases, we assessed whether HOPS shuttling was sensitive to growth factors. As many researchers assessed the role of growth factors in driving lens differentiation [14,15], we injected EGF in mouse in order to evaluate HOPS localisation.

**Figure 4:**
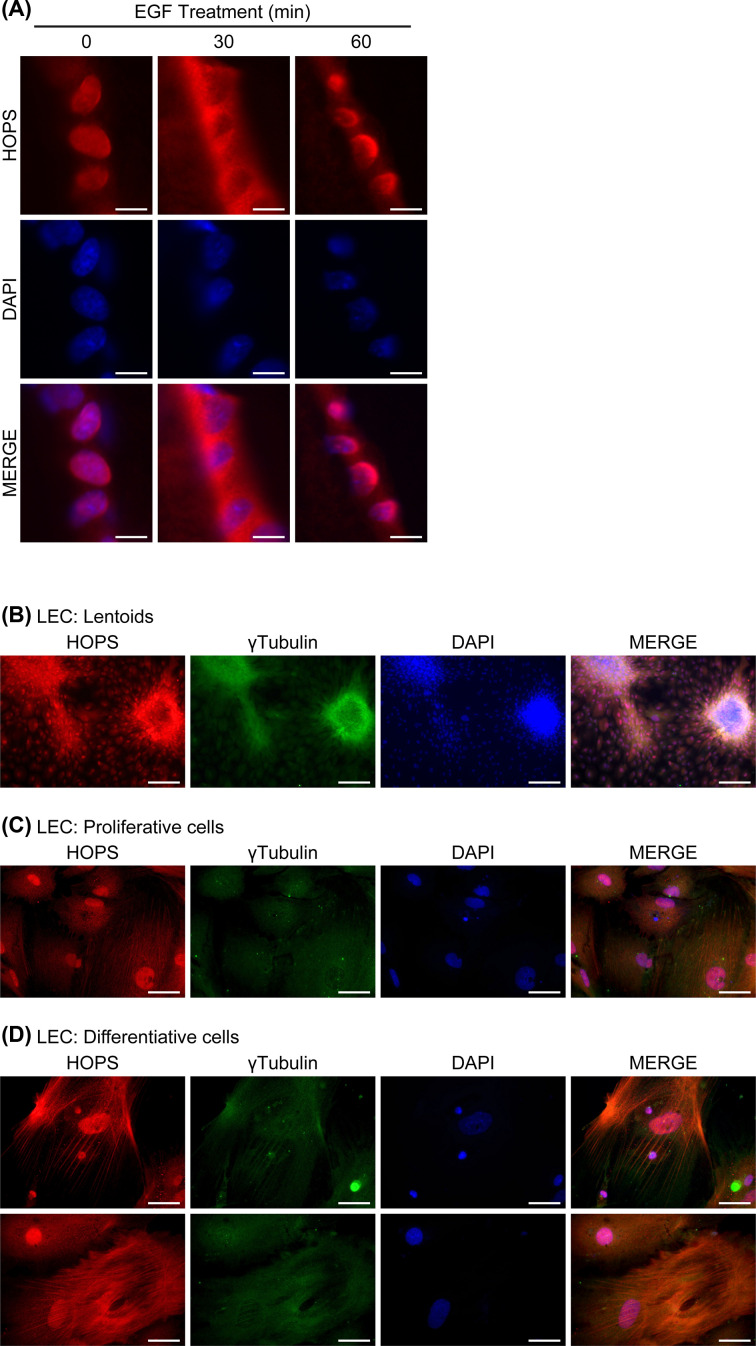
HOPS localisation in LEC (**A**) HOPS (red) nucleo-cytoplasmic shuttling was observed after EGF treatment. Nuclei were DAPI stained (blue). Bars, 5 μm. (**B–D**) HOPS localisation (red) in differentiating lens primary cell cultures mimicking *in vivo* development. HOPS immunolocalisation was analysed in lentoid (B), proliferating (C) and differentiating cultured cells (D). Nuclei were DAPI stained (blue). γ-tubulin (green) was used as marker. Bars, 200 μm in (B) and 5 μm in (C,D). Merged images are reported. Representative images are shown.

Of note, just 30 min after injection, EGF treatment induced HOPS migration to cytoplasm even in CZ, showing a dynamic and sensitive HOPS shuttling in proliferating cells arrested among exposure to EGF. Sixty minutes after EGF inoculation, HOPS has moved back to nucleus (Figure 4A).

### HOPS in the lens primary cell culture

To evaluate HOPS behaviour during differentiation, we examined HOPS localisation in the lens primary cell cultures. The cell culture is able of mimicking lens development as it occurred *in vivo*, forming lens-like structures known as lentoids. Lens primary cell cultures show many biological characteristics similar to the *in vivo* differentiated cells of the lens. Use of primary cell cultures allowed to observe lens cell differentiation *in vitro* [25].

To this end, we prepared lens primary cells from mice eye explants. We induced cells to differentiate by adding FGF to the culture medium. Keeping cells in culture with the same culture medium for several days, differentiation areas were identified by the presence of the lentoid bodies (Figure 4B) [26].

HOPS localisation was observed in both proliferating and differentiating cells of LECs using γ-tubulin as marker [27]. In proliferating cells, far from lentoids, HOPS was revealed both in the cytoplasm and in the nucleus depending on the cell cycle phase (Figure 4C). Instead, in differentiating cells, HOPS was associated to the lens fibers. These results agree with the *in vivo* data (Figure 4D).

## Discussion

The lens is a simple experimental model to study cellular processes in a single structure, presenting simultaneously cells in quiescence, proliferation and differentiation state. Indeed, all these events are well separated in distinct lens compartments.

As previously published, we describe HOPS as a shuttling protein that moves from nucleus to cytoplasm depending on cell cycle phases both in regenerating liver and tumour cells [2]. Recently, we highlighted that HOPS ability to act as tumor suppressor not only relies on its expression, but also on its localisation. In nucleus HOPS overexpression stabilises p19^Arf^, which in turn activates p53 that arrests the proliferation [4]. Interestingly, similar results have been observed in lens. Here, we show for the first time HOPS in the nucleus of quiescent epithelial cells of CZ, while in GZ and TZ—proliferative and differentiative regions respectively— HOPS is prevalent in cytoplasm. Since now no results have been published about HOPS localisation in differentiating cells. The lens offers an excellent model to study HOPS localisation in differentiation. Our results indicate that during cell differentiation HOPS shuttles from cytoplasm to nucleus, inducing proliferative arrest and starting differentiation. These data agree with the results obtained *in vitro* using cultured LECs where the progressive accumulation of HOPS in nucleus is accomplished with cell differentiation.

An important role in controlling proliferation and differentiation is played by p53. Indeed, p53 controls proliferation by activating p21 [28], and differentiation by triggering apoptotic signalling in the programmed removal of organelles from differentiating lens fiber cells [29].

This apoptotic program is fundamental to sustain lens transparency through formation of an organelle-free zone. We can speculate that HOPS localisation in nucleus and the differentiation of lens cells are accompanied by a progressive arrest of proliferation, which triggers the differentiation. Recently, we demonstrated that HOPS UBL plays a notable role, as protein modifier, regulating p53 stability and triggering the p53-mediated apoptosis [5]. In such a complex structure as lens is the role of protein modifiers and ubiquitin is fundamental and the machinery involved in proteasomal pathway execution is strategic in the shift from proliferation to differentiation [30,31].

In the present study we demonstrate that HOPS is engaged in regulating proliferation and differentiation of the lens texture having a possible role, as UBL modifier, in the final constitution of the differentiated structure of the lens. Together with the crystallin superfamily and the other lens proteins, HOPS may contribute to warrant the transparency of the structure by regulating various proliferative or apoptotic events to avoid the cataract formation [10,32].

## Data Availability

The authors agree to make any materials, data, code and associated protocols (relating to their published research) available to *bonafide* researcher or reader requests without undue delay or qualifications.

## Abbreviations

BrdU: bromodeoxyuridine
CR: central region
CZ: central zone
DAPI: 4′,6-Diamidino-2-Phenylindole, Dihydrochloride
EGF: epidermal growth factor
GZ: germinative zone
HOPS: hepatocyte odd protein shuttling
LEC: lens epithelial cell
OCT: optimum cutting temperature
PH: partial hepatectomy
qPCR: quantitative PCR
TMUB1: trans-membrane ubiquitin-like containing protein 1
TZ: transitional zone
UBL: ubiquitin-like domain
